# Corporate capital structure effects on corporate performance pursuing a strategy of innovation in manufacturing companies

**DOI:** 10.1016/j.heliyon.2024.e24677

**Published:** 2024-01-25

**Authors:** Fahad Ahmed, Mujib Ur Rahman, Hafiz Mudassir Rehman, Muhammad Imran, Anna Dunay, Md Billal Hossain

**Affiliations:** a School of Information Technology, Washington University of Science and Technology, VA 22182, USA; b Faculty of Business & Economics, Abdul Wali Khan University Mardan, 23200, Khyber Pakhtunkhwa, Pakistan; c Department of Global Business & Enterprise, Ulster University Business School, Ulster University, BT487JL, UK; d Faculty of Administrative & Management Sciences, Khwaja Fareed University of Engineering and Information Technology (KFUEIT), Rahim Yar Khan, Punjab, Pakistan; e Doctoral School of Management and Business Administration, John von Neumann University, 1117 Budapest, Hungary; f Business Management and Marketing Department, School of Business and Economics, Westminster International University in Tashkent (WIUT), Tashkent 100047, Uzbekistan

**Keywords:** Corporate capital structure, Debt finance, Economic growth, Money and interest rates

## Abstract

Within the sphere of finance, the concept of capital structure has long been a subject of intense debate, serving as a quantitative depiction of the balance between debt, preference shares, and common stock within a company. This structure serves a crucial role in optimizing the utilization of a company's existing resources while simultaneously elevating the revenue streams for stakeholders. This particular study delves into the intricate relationship between corporate performance and capital structure, focusing on 78 publicly listed firms within the Dhaka Stock Exchange (DSE). Bangladesh holds the 29th position globally in terms of purchasing power, lending significant weight to this investigation. To comprehensively analyze this correlation, panel data encompassing the span from 2017 to 2021 was collected for these 78 sample companies operating within the DSE. Several key determinants of capital structure were considered in this analysis, namely the debt-to-equity ratio, short-term leverage ratio, long-term leverage ratio, and total debt ratio. Meanwhile, the performance of these firms was gauged using key metrics such as Return on Assets (ROA), Return on Equity (ROE), and Earnings Per Share (EPS). To ensure a robust analysis, factors such as inflation, liquidity, growth rate, tax rate, and firm size were meticulously controlled for. The findings unveiled a compelling narrative: all forms of debt ratios—be it short-term, long-term, or the total debt ratio—exhibited a substantial negative impact on ROA at a significant level of 1 %. Conversely, specific debt ratios, like the short-term total debt and the total debt-to-total asset ratio, displayed a notable positive correlation with ROE at a 1 % significance level. Intriguingly, the long-term total debt ratio yielded a negative and insignificant effect on ROE. Moreover, within the spectrum of predictors influencing a firm's performance, the liquidity ratio emerged as a non-significant factor—a notable discovery that highlights the nuanced nature of the interplay between capital structure and performance within these companies.

## 1 Introduction

Capital structure is a long-debated issue in the field of finance. When it comes to the decision for the financial aspects or performance, capital structure is given the priority as it is a way to finance a company with a combination of equity and debt to obtain maximum profit and optimal capital structure [1]. A corporate capital structure is the many ways it raises money for its daily operations and future growth. It is a numerical representation of a firm's balance sheet's debt, preference shares, and common stock weights. Long-term debt to equity ratio defines a company's financial structure [2]. The firm's capital structure may be used to calculate its long-term debt-to-equity ratio. Debt and equity capital, two types of finance, must be handled separately [3]. An organization's management team's ability to create reliable financial assessments may be used to assess its finances. Think about a company's return on investment, return on equity, earnings per share, dividend yield, price to earnings ratio, sales growth, market capitalization, and so on [4]. Despite the fact that the findings of an analysis into the relationship between financial decisions and performance were inconsistent, it is still necessary to determine whether or not the capital structure of a company has an effect on the success of the company. In addition, the vast majority of empirical research on this topic has been carried out in developed financial markets, while just a select few empirical studies have been carried out in developing financial markets like Bangladesh [5]. This indicates that it is of the utmost importance to investigate the capital structure of the Dhaka Stock exchange (DSE) and the connection that it has with company performance [6].

But when there is no internal funding, the trade-off theory represented that the firm financed both debt and equity to avoid risk and obtain optimal capital structure. Besides, the tax advantage of debt, the cost of financial distress, including non-bankruptcy costs and bankruptcy costs, forces firms to choose between debt and equity to obtain optimal capital structure [7]. According to Vătavu [8], the choice of debt and equity financing has a remarkable influence on the operation and profitability of the business. Extending the viewpoint [9], focused on the significant impact that capital structure has for financial performance while shaping the activities. Because the optimal capital structure ensures the required level of capital at the lowest weighted average cost of capital. Which, in the long run, increases the firm's value. Kyereboah-Coleman [10] identified the relationship and significance between capital structure and financial profitability. They opined that organizations should focus on setting capital structure in such a way that it helps to maximize shareholders' wealth. Similar studies conducted by Anowar [11], Joghee et al. [12] and Rakesh & Souza [13] also show that the capital structure has a remarkable influence on financial profitability and performance.

There are only a few researchers considered the Dhaka stock exchange, such as Anowar [14], Sayeed [15], Rouf [16], and Chowdhury [17]. The research conducted by Anowar [11] on Bangladeshi firms considered ROA as a profitability indicator where Rouf [18] considered both ROA and ROS to analyses the effect of these profitability indicators on capital structure proxies. But both Anowar [14] and Rouf [18] did not consider ROE, which is another most essential factor in analysing the impacts of the capital structure on the development of financial performance. Moreover, the research of Anowar [11] considered only firm size, current ratio, and long-term debt to assess the effect of capital structure on firm profitability [19]. There are so many capital structures proxies and firm performance proxies that were not considered in previous research. Besides, some studies are lack of small amount of variable or small data period. The research of Rouf [18] conducted on DSE non-financial firms considered ROA and ROS to assess the impacts that capital structure has on a firm's financial performance. Moreover, he took only 4 years of data. But our study includes 5 years of latest data and ROA, ROE, and EPS as profitability indicators. Our research increases the number of firms to assess the impacts that capital structure has on a firm's financial performance [20,21].

### 1.1 Research questions

This research paper is looking to answer the following questions.

i. How a firm's capital structure and its elements influence financial performance?
ii. What is the nature of relationship between capital structure and corporate financial performance of the selected companies in Bangladesh?
iii. What are the element of the capital structure which has the most level of influence and impacts on developing or reducing financial performance?
iv. How the companies should select their capital structure to utilize the cost-saving sources of funding for increasing the firm's value in the long run in Bangladesh?

Bangladesh was chosen because of its developing market economy, which places it at number 29 globally in terms of purchasing power. With a real GDP growth rate of 7.3 %, it is also the world's seventh-fastest growing economy and one among the next eleven emerging middle-income economies. Additionally, the government has just recently authorised the establishment of 37 new economic zones (including governmental, non-governmental, and Specialized Economic Zones) to attract investors from all over the world. As a result, it is important for investors to gain knowledge while pinpointing the effects of various debt uses and the capital structure proxies that matter most for business success in Bangladesh. Investors have a duty to do this in order to meet their obligations.

The outcome of this research is different from previous research in many aspects. Because in previous research on DSE, they did not include very important control variables such as inflation rate, gr rate, tax rate, liquidity, and firm size, which have a very significant effect on ROA, ROE, and EPS according to Vatavu [22]. This paper contributes to the prior literature in Bangladesh by including both market proxy EPS and ROE, which is a crucial factor in firm performance analyses. Besides, our research is conducted by including a recent long period from 2017 to 2021, increasing the number of firms and finally including Dependent variable ROE, EPS, and control variables such as liquidity, inflation rate, firm tax, gr_rate, and firm size. Finally, this research divided the regression model into five different parts so that it can find out each independent variable effect on firm performance more precisely.

### 1.2 Objectives of the study

• To Assess the Impact of Capital Structure on Financial Performance:
• To Examine the Nature of Relationship between Capital Structure and Financial Performance.
• To Identify Key Elements Influencing Financial Performance within Capital Structure.
• To Recommend Optimal Capital Structure for Long-term Firm Value Enhancement.

### 1.3 Significance of the study

The quest to discern the precise threshold of debt beyond which firms experience a decline in value remains a critical gap. Understanding the nuanced trade-offs between the benefits of tax-saving from debt and the costs associated with bankruptcy is essential. The varied outcomes observed in different countries and industries regarding the impact of debt on firm performance underscore the need for more contextualized analyses. By delving deeper into localized economic contexts, this study intends to unravel the nuanced relationships between debt structures and firm performance indicators across diverse sectors. The dynamic nature of the relationship between debt and firm performance necessitates a more comprehensive understanding of how this link evolves over time, especially amidst fluctuating economic cycles. This study aims to explore the temporal dynamics of debt's influence on firm performance across varying market conditions. Recognizing the significance of non-debt factors such as liquidity, growth rate, inflation, and firm size in shaping firm profitability, this study seeks to elucidate their interplay with debt structures. Understanding how these factors collectively impact firm performance forms a pivotal part of this investigation.

## 2 Literature review

According to the [8,78] famous MM-theorem, that in a tax-free market, the capital structure does not affect the value and performance of the firm, which is known as the proposition of the M&M theorem of capital structure. However, the second theory of Miller and Modigliani states that when corporate tax is considered, using debt increases the firm value because the interest cost of debt in the company is tax-deductible based on its significance. Thus, this reduces the expense and ultimately increases firm value [23,24]. So, relying on debt financing, organizations can increase their value. However, the firm should not use debts beyond a certain level because it creates a risk of bankruptcy. Ross [25] has also shown in his research that when debt level increases, it provides a positive signal in the market as investors think that an organization can take debt and repay it where a decreased level of debt provides terrible signals. However, Acharya, Richardson [24] argued that a high level of debt creates the unavailability of the fund because it makes the organization looks vulnerable and risky to the lenders [23]. Besides, it increases the cost of debt, and thus, the ability to take debt at favourable terms becomes hard.

Miller extended the MM theory and developed a new theory called the trade-off theory. For the capital structure, this Trade-off theory imposes that the company chooses between a balance of the deadweight cost of bankruptcy and the tax-saving benefit of debt. As MM perfect market is absent in real-world, the firm which uses more debt has more chance to fall in financial distress. Stiglitz [26] stated that a firm value reaches high when marginal bankruptcy costs and advantages of marginal tax shield costs become equal. Similar results may be seen in investigations by Warmer [27] and Altman [28]. They also found that when a specific threshold was reached, the likelihood of suffering financial difficulties increased as the amount of debt increased. They saw this first hand. Costs incurred by the bankruptcy process reduce the value of the firm and, by extension, the value to the creditors. After doing the math, Kim [29] concluded that the optimal capital structure might be attained if interest income were to equal the present value of expected bankruptcy costs. This result stems from the research they conducted. As a result, the Trade-off theory is a concept that recommends reaching the optimal capital structure by finding a happy medium between debt and equity [30].

Aggarwal & Gupta [31] observed the effect of Dequity on firm performance in Bangladesh and found that Dequity was negatively at 1 % significant with both firm performance indicator ROA and ROS. However, Saputra, Noer, and Lukytawati [32] investigated the impacts that capital structure had on a firm's financial performance on the financial institution in Indonesia from 2009 to 2013. They used ROA and ROE as the dependent variable. They divided the data into different subsectors such as Banking, Funding, Securities, Insurance, and others. They found that only in the Funding subsector Debt to equity ratio was at 5 % negatively significant with ROA. Security subsector Debt to equity was at 1 % positively significant with ROE. However, on other subsectors, there was no relationship between Debt-to-equity ratio and ROA and ROE. Indonesian financial institution used more leverage than their internal funding. With increasing, debt level firm value increased at a certain level after that firm value decreased with increasing Debt [33]. Which overall effect we could see in the Indonesian financial institution.

On the other hand, Ramachandran analysed the effect of capital structure on firm performance by dividing Indian IT firms into three sectors and these were low-income firms, medium-income firms, and high-income firms. He found that Dequity was negatively significant at 1 % with ROA in terms of medium income and high-income IT firms. The findings revealed that Debt in medium-income and high-income IT firms reduced their net profit significantly. So, they had an inverse relationship between Dequity and ROA. This result is similar to ([34]; Huang and song 2006; [35,36]) while dissimilar with Sayeed [15] and Rub [37].

Panda, & Nanda [38] found a significant negative relationship between Short-term debt (STD) and profitability when ROE measured profitability. Because at the time Jordan economy downturn, there seems to cash inflow problem. As a result, firms start liability payments, and default risk increased. Vatavu [22] analysed the relationship in Romanian manufacturing companies. He showed that there was a significant negative relationship at 1 % between STD and both ROA and ROE. He stated that the Romanian company could be more profitable by decreasing short term debt and increasing the high proportion of equity. However, Elena Alexandra & Georgeta Vintilla [39] also showed that there was a negative association between STD and ROA in the Romanian company between the year 2000–2016 [40].

Ikapel and Kajirwa [41] investigate the relationship between long term debt of the companies and financial performance in Kenya during 2004–2014 indicated by ROA. He found that LTD was negatively statistically insignificant (p > 0.05) with ROA. Because profitable companies preferred internal funding rather than Debt.

Khanna, A., & Puri, B []. found in his research in Romanian company from 2003 to 2010 that liquidity had a significant positive effect on ROA, and ROE at 1 % means it helps the lenders to assess the financial position of the organization as it is the mark of solvency. Thus, it indirectly affects the cost of capital and, thus, profitability. He used ROA and ROE as his dependable variable. Following this, Rouf [16] investigated the effect of liquidity on firm performance in Bangladesh and found a positive but no significant relationship between them.

Research conducted by Musah [42] on 23 banks of Ghana found that liquidity negatively affects the profitability of those banks. Strebulaev [43] also found a negative relationship between liquidity and profitability because, according to trade-off, dynamic theory profitability has a negative correlation with liquidity. Besides, Anowar [44] found a unidirectional causality between liquidity and capital structure means those firms use less external Debt. However, DR. D K Y Abeywardhana [45] and Booth et al. [46] also showed that liquidity had a negative effect on all measures of performance.

Chada & Sharma [47] argued that a firm with a high growth rate could choose short term debt for the company or the capital instead of long-term debt to reduce their agency cost in the market. Finally, it helps to improve their profitability [48]. The research of Martis [49] conducted on S&P 500 firms of U.S found gr_rate as a factor that had a positive relationship with ROA and ROE.

The gr_rate increases the profit of the organizations and thus increases profitability. The research conducted on Bangladeshi firms by Akhter et al. [23] also found a positive relationship between gr_rate and profitability of an organization.

Ajayi and Zahiruddin [50] investigate the relationship between capital structures on firm performance in Nigeria from 2010 to 2014 with 100 non-financial firms. They found a significant positive relationship between sales and firm performance. The author described an increase in sales leads to an increase in financial performance. However, Rouf [16] in Bangladesh, Abeywardhana in the U.K., and Mahfuzah [51] showed that gr_rate has a significant positive effect on firm performance [52].

Khanna, & Puri [] argued that inflation of an organization is a macroeconomic factor that influences the performance of firms as inflation reduces the value of money. He found that the inflation rate had a very significant positive effect on ROA at 1 %. Roberts et al. [53] found a negative relationship between firms’ performance and inflation. They also stated that inflation reduced the value of the return provided by the organizations, and hence negative relationship pertains. The research conducted on U.S. S & P 500 firms by Martis [49] found the inflation rate as a significant factor influencing ROA. But he did not find tax as a factor that influences the ROA of the U.S. firms and stated the reduction in the purchasing power reduces the value of money the stakeholders receive as a return [54]. Thus, a negative relationship exists with profitability. Maria Rashed Awan investigated the effect of inflation rate on firm profitability in 2014 in Pakistan from 2006 to 2011. She found a very positive significant result between Inflation rate and firm profitability [55].

The size of the firms affects profitability because of the source of capital used to expand the operation of the firms [53]. The research on Mishra, & Padhi [56] on S&P found a positive relationship between firm size and profitability. This is because larger firms have more opportunities to grow and have the ability to negotiate with their suppliers to buy materials at a lower price which ultimately creates the opportunity to increase profitability.

Shubita & Jaafer [57] in their extending studies depicted a positive relation between firm size and firm performance. Similarly, Ajayi and Zahiruddin [58] tested whether firm size had any impacts or positive impacts that capital structure has on the firm's financial or not. They used ROA and Tobin's Q as a proxy for firm performance. They got a very significant positive relationship between ROA and Firm size at 1 %. However, Boone et al [59] described that outside director and firm size had a positive relation means larger the firms the larger the number of the director will be. Which will help the firm in the quest for transparency and efficient monitoring overall improve firm performance. Anowar et al. [60] researched Bangladeshi firms and opined that Bangladeshi firm prefers leverage to increase its firm size and this ultimately increases the interest expense as well as increases the cost of capital. So, the excess leverage decreases the ROE when the firm enhances its size using a high level of debt.

The research of Dong et al. [61] referred to Miller and Modigliani proposition II, which suggested the tax shield advantage from using Debt and hence, a positive relationship found between tax and profitability measures. Besides, Serfling [62] also found a highly positive correlation between tax and ROE as the increased level of tax reduced the interest expense of Debt, and this could be a reason to increase profitability. Cheng and Tzeng [63], however, argued that the tax level also increased the personal tax of the shareholders; thus, the tax savings from the interest payment net off at the individual level. Martis [64] researched S & P 500 firms to assess the significant impacts of capital structure on firm's success and profitability and did not find any significant influence of tax on profitability.

### 2.1 Research gap

The extensive literature review on the relationship between capital structure and firm performance reveals several compelling insights. While numerous studies have explored the impact of debt on firm value, a critical gap lies in identifying the precise threshold beyond which increased debt levels lead to a decline in firm value. Although the trade-off theory suggests an optimal balance between the costs of bankruptcy and tax-saving benefits, defining this threshold remains elusive across different industries and economic contexts. Variations in the impact of debt on firm performance across different sectors and countries highlight a need for more context-specific analysis. The existing studies from Bangladesh, Indonesia, India, and other countries exhibit divergent results concerning the relationship between debt-to-equity ratios and financial performance, emphasizing the need for localized and industry-specific research. The dynamic nature of the relationship between debt structure and firm performance requires further exploration. The existing studies have largely focused on a specific timeframe, lacking in-depth analysis of how this relationship evolves over varying economic cycles or periods of market instability. While debt's influence on firm performance has been extensively studied, there is a relative paucity in understanding the interplay of non-debt factors, such as liquidity, growth rate, inflation, and firm size, on firm profitability. Research gaps persist in delineating how these factors interact and jointly impact firm performance. The relationship between taxation, particularly the tax shield from debt, and firm profitability warrants further investigation. Conflicting findings exist regarding the impact of tax on profitability, emphasizing the need for more nuanced exploration of tax implications on firm performance. Although several studies have examined the relationship between firm size and profitability, the contextual influence of firm size across different industries and geographical locations remains an under-explored area.

### 2.2 Research hypothesis

Hypothesis 1 There exists a significant relationship between a firm's capital structure and its financial performance.

Hypothesis 2 The relationship between capital structure and corporate financial performance among the selected companies in Bangladesh will exhibit a notable correlation.

Hypothesis 3 Certain elements within the capital structure impact a higher level of influence in either enhancing or diminishing financial performance within the selected companies in Bangladesh.

Hypothesis 4 The selection of an optimal capital structure by companies in Bangladesh, geared towards cost-saving funding sources, will positively impact the long-term firm value.

## 3 Research methodology

### 3.1 Data collection method

There are 586 listed organizations in the Dhaka Stock out of which 369 are financial companies. As a source of data this research excluded all 369 the financial companies as all the companies which were establish after 2014, and the companies which had missing variable. Finally, this research considers a sample of 78 companies to collect numeric information from their published annual reports on the website of Dhaka Stock Exchange. A similar model has been adopted in several studies [65,66].

### 3.2 Source of data

This study is based on 78 firms in pharmaceuticals & chemicals, Fuel & Power, engineering, ceramics, and IT, which are listed in the Dhaka Stock Exchange until 2020. Among 78 firms, engineering has 36 firms, Pharmaceuticals & Chemicals Industry has 28 firms, Fuel & Power has 18 firms, Ceramics has 12 firms, the IT industry has 8 firms, Cement Industry has 7 firms and Ceramics industry has 5 firms. This research collected panel data from 2017 to 2020 from the annual reports of the company.

The published annual reports of these companies were the source of the collected data. And the year-end of these financial reports was 31st December. Finally, the control variable i.e., data regarding gr-rate, inflation rate, and tax, have been collected from the Bangladesh Statistic Bureau website. After collecting the panel data of these sample firms from the annual reports, a descriptive analysis has been conducted.

### 3.3 Dependent variables

In this study $Y_{i}$ is the dependent variable represent Financial Performance or Profitability measured by Return on Asset (ROA), Return on Equity (ROE) and EPS (Earning per Share).

### 3.4 Independent variables

The independent variables are long-term debt (LTD), short-term debt (STD), total debt ratio (TDR), debt-equity ratio (Dequity), liquidity, growth (gr-rate), inflation rate (Inf-rate), Neutral log value of tax (ln-tax) payment, firm size.

## 4 Research model

The research model employed in this study aims to investigate the impact of capital structure on financial performance by utilizing a regression equation encompassing three dependent variables and a multitude of independent and control variables. The examination is rooted in panel data analysis covering 78 firms across the years 2017–2021. To determine the most suitable panel data analysis model, the Hausman test has been administered.

This research model draws inspiration from previous empirical studies conducted by scholars such as Vatavu [22], Rouf [16], Abeywardhana [45], Ramachandran and Gavoury [67], and Mahfuzah & Yadav [68]. These studies have extensively explored factors influencing firm performance across various countries. The adoption of this model is substantiated by its widespread usage among researchers, employing Return on Assets (ROA), Return on Equity (ROE), and Earnings Per Share (EPS) as dependent variables to gauge firm performance. The independent variables considered encompass long-term debt, short-term debt, total debt ratio, debt-equity ratio, liquidity, growth rate, inflation rate, tax, and firm size.

An observation derived from the Pearson correlation table highlights significant correlations among certain independent variables, specifically noting strong positive correlations between Dequity and STD, LTD, and TDTA at a significance level of 1 %. Similarly, TDTA demonstrates a robust correlation with Dequity, STD, and LTD at a 1 % significance level. Moreover, STDA exhibits a substantial correlation with TDTA. These strong correlations between independent variables raise concerns about multicollinearity, potentially impacting the validity of the results derived from a single research model.

To mitigate the issue of multicollinearity and derive valid and reliable conclusions, the study opts to analyze the effect of each independent variable on firm performance separately. Consequently, the decision has been made to construct separate models for each independent variable, allowing for a more nuanced examination of their individual impacts on firm performance. This approach aims to yield more accurate and insightful findings, steering clear of potential distortions caused by multicollinearity issues within a single comprehensive model.

Model 1 This regression model will represent and evaluate the relationship between Dequity and a firm's performance

$$Y_{i}=\alpha +{\;\beta }_{1}\mathrm{dequity}+{\;\beta }_{5}\mathrm{liquidity}+{\;\beta }_{6}gr\_rate+{\;\beta }_{7}\;\inf\_\mathrm{rate}+{\;\beta }_{8}\;\ln\_\mathrm{tax}+{\;\beta }_{9}fm\_size+e$$

Model 2 The relationship between STD and firm's performance is tested by the following.

Regression model

$$Y_{i}=\alpha +{\;\beta }_{2}\;\mathrm{STD}+{\;\beta }_{5}\mathrm{liquidity}+{\;\beta }_{6}gr\_rate+{\;\beta }_{7}\;\inf\_\mathrm{rate}+{\;\beta }_{8}\;\ln\_\mathrm{tax}+{\;\beta }_{9}fm\_size+e$$

Model 3 The relationship between LTD and firm's performance is tested by the following.

Regression model

$$Y_{i}=\alpha +{\;\beta }_{3}\mathrm{LTD}+{\;\beta }_{5}\mathrm{liquidity}+{\;\beta }_{6}gr\_rate+{\;\beta }_{7}\;\inf\_\mathrm{rate}+{\;\beta }_{8}\;\ln\_\mathrm{tax}+{\;\beta }_{9}fm\_size+e$$

Model 4 The relationship between TDTA and firm's performance is tested by the following.

Regression model

$$Y_{i}=\alpha +\beta 4TDTA+{\;\beta }_{5}\mathrm{liquidity}+{\;\beta }_{6}gr_{\mathrm{rate}}+{\;\beta }_{7}\mathrm{in}f_{\mathrm{rate}}+{\;\beta }_{8}ln_{\mathrm{tax}}+{\;\beta }_{9}fm_{size}+e$$

Model 5 The relationship between STD and LTD with a firm's performance is tested by the following Regression model

$$Y_{i}=\alpha +{\;\beta }_{2}\;\mathrm{STD}+{\;\beta }_{3}\mathrm{LTD}+\beta {\;\beta }_{5}\mathrm{liquidity}+{\;\beta }_{6}gr\_rate+{\;\beta }_{7}\;\inf\_\mathrm{rate}+{\;\beta }_{8}\;\ln\_\mathrm{tax}+{\;\beta }_{9}fm\_size+e$$

Where, $Y_{i}$ is the Financial Performance or Profitability measured by Return on Asset (ROA), Return on Equity (ROE) and EPS (Earning per Share).

## 5 Results and discussions

The descriptive analysis for the sample of Bangladeshi firms are given in table-1. ROA has mean, minimum, and maximum of 0.075, −0.15 and 0.747respectively. ROA is one of the major indicators of firm performance, so the higher the returns from assets, the better the firm would perform. ROE statistics show 0.152 mean, 1.727 maximum, −0.24 minimum, and 0.010 standard deviations. In terms of ROE, Bangladeshi companies seem to generate profit from their shareholders as ROE value is not below Zero. On the other hand, EPS has a data range from 0.020 to 9.82, with a 3.092 mean.

**Table-1:** Descriptive statistics of the variables.

Variables	Mean	Standard Error	Median	Minimum	Maximum
Dequity	1.3559	0.1088	0.7303	−2.6676	17.5300
STD	0.3566	0.01414	0.3104	0.0124	2.7083
LTD	0.1257	0.0091	0.0695	0.0004	1.6035
TDTA	0.4599	0.0141	0.4307	0.0132	1.8157
liquidity	2.3560	0.2265	1.5942	0.0066	76.1325
Gr_rate	0.1502	0.0244	0.0918	−0.9992	4.1590
Inflation Rate	0.0639	0.0004	0.0616	0.0561	0.0750
Ln (Tax)	17.5004	0.1188	17.7203	10.2587	21.9844
Firm Size	22.1354	0.0802	22.1844	18.0963	25.7863
ROE	0.1522	0.0101	0.1020	−0.2428	1.7456
ROA	0.0754	0.0049	0.0492	−0.1507	0.7475
EPS	3.0925	0.1177	2.6600	0.0200	9.8200
N			377		

*Estimated over 5 years, 2017–2021.

Debt-equity ratio (Dequity) has a mean, minimum, maximum, and standard deviation of 1.35, −2.67, 17.53, and 0.11 respectively indicate that Bangladeshi company used borrowed funds more than internal funds in their fixed asset investment. Liquidity statistics 2.35 mean, 76.133 maximum, 0.006 minimum, and 0.23 standard deviation mean that current asset proportion is too large, and temporary debt can cover half. The average mean of STD or short-term debt-total asset ratio, LTD or long-term debt-total asset ratio and TDTA or Total debt to total asset ratio are 1.35, 0.35, 0.12 and 0.45 respectively. This indicates that the Bangladeshi company prefers short term debt than long term debt. A similar result was found in the research of [55], where it has been stated that Bangladeshi listed firm has higher short-term debt in their capital structure than long term debt.

These presumptions based on a supposed linear connection. A straight line that is horizontally oriented and devoid of patterns is an example of a linear connection. Since the residual plot shows no clear trend, this provides support for this conclusion. This suggests that a linear relationship may be inferred between the independent and dependent variables.

The QQ plot of the residuals allows for a visual inspection of the normality assumption. A straight line should appear in a probability plot of residuals if everything goes according to plan. All the data points cluster close to this common center.

**Fig. 1:**
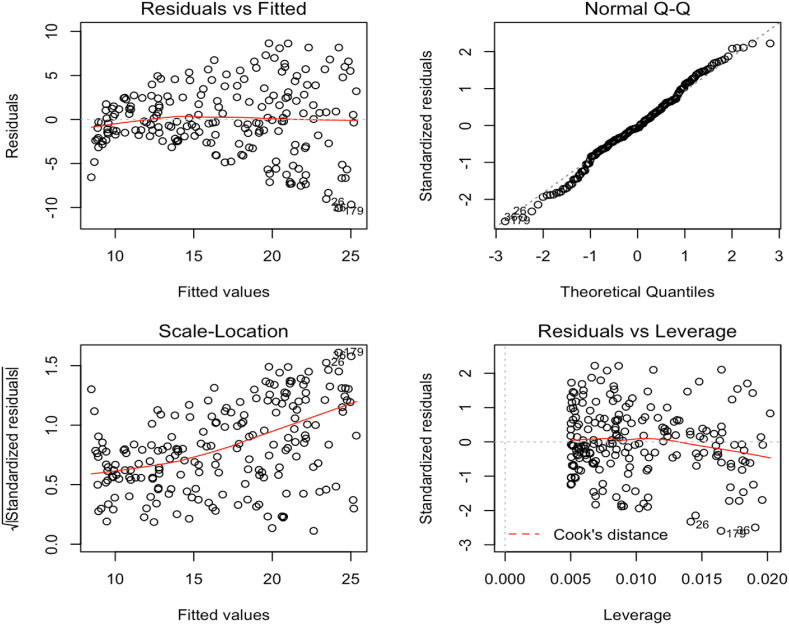
Standardized residuals.

Standardized residuals below −2 are shown (Fig. 1) graphically for the three most severe cases. Coordinates (26, 36, and 179) are where these places are found. On the other hand, no extreme values outside the mean occur, which is a positive sign.

In addition, it seems that the dataset lacks a key area of leverage. In other words, the leverage statistic for each data point is less than 2 (p + 1)/n, where p is the number of predictors and n is the size of the sample. The relevant cutoff in this instance is 4/200, or 0.02.

The correlation matrix in table-2 has shown the negative relationship between Dequity and liquidity, although the correlation (−0.13) is at 5 % significant. The negative relationship has been developed between STD and liquidity (−0.27371) at a 1 % level of significance but moderately positive correlation i.e., at a 5 % level pertains between STD and Dequity (0.39). The LTD also has a negative relationship with liquidity (−0.042). This is natural because short-term debt organizations used to take when they fall short of cash or other liquid sources to conduct the financial, according to [55,61].

**Table-2:** Pearson correlation matrix.

	Dequity	STD	LTD	TDTA	liquidity	Sales Gr	Inf_rate	Ln (Tax)	Firm Size	ROE	ROA	EPS
**Dequity**	1											
**STD**	0.398**	1										
**LTD**	0.144**	−0.015	1									
**TDTA**	0.535**	0.518**	0.609**	1								
**liquidity**	−0.132*	−0.27**	−0.043	−0.27**	1							
**Sales Gr**	0.016	−0.017	0.069	0.032	0.058	1						
**Inf_rate**	−0.014	−0.027	−0.025	−0.055	0.084	0.054	1					
**Ln (Tax)**	0.148**	0.108*	0.044	0.055	−0.158**	−0.039	−0.011	1				
**Firm Size**	0.196**	0.009	0.121*	0.048	−0.068	−0.023	−0.061	0.752***	1			
**ROE**	0.212**	0.204**	−0.048	0.157**	−0.085	0.086	−0.063	0.251**	0.069	1		
**ROA**	−0.119*	0.042	−0.19**	−0.16**	−0.020	0.082	−0.052	0.239**	0.011	0.885**	1	
**EPS**	0.061	0.211**	−0.053	0.108*	−0.124*	0.0189	−0.078	0.488**	0.326**	0.348**	0.330**	1

**And * indicate statistical significance at 1 % and 5 % level, respectively.

The pecking order theory also suggests that when an organization has enough liquid assets, it tends to use liquid asset to conduct regular operating activities. The research of Rouf [16] also found a significant negative relationship between liquidity and leverage. According to the trade-off theory, organizations with a high level of liquidity tend to have a greater ability to meet short-term debt obligations.

According to the findings of this study, there is a connection between TDTA and Dequity, STD, LTD in Bangladeshi firms quite strong, having the co-efficient 0.534, 0.52, and 0.61respectively, which significance level is more than 5 %. Moreover, we have found a negative relationship with liquidity and all other variable considered in the model except ROA. However, the relationship, whether positive or negative, between liquidity and other variables is not that strong. ROE has been expected to be positive but it turned out to have a negative relationship with LTD and liquidity. The coefficients are −0.047 and −0.084, respectively. Even if the relationship is not that strong, the research of Ismail [69] stated that ROE tends to have a negative relationship with LTD because increasing long-term debt instruments creates an obligation to provide interest, and thus, this reduces the return to the equity holder to some extent (Dong et al. [61].

The correlation coefficient between ROA and ROE is 0.88, which indicates that there is a strong positive link between the two metrics. A similar result was found in the study conducted by Anowar [11], where the research considered over 62 DSE listed manufacturing firms. The more the return on asset, the more return the equity holders get. Also, Eps is highly correlated with ROE and ROA, respectively 0.35 and 0.33 at a 1 % level. However, ROE has a negative relationship with LTD (−0.047) because an increased level of debt usage shrinks the return available to the equity holder, and hence, this reduces ROE. A similar result was found in the empirical research conducted by [55,11], considering 40 manufacturing firms of Bangladesh in 2016 and found a significant and growing negative relationship between ROE and LTD. He opined that the more long-term debt in the capital structure, the more fixed interest cost increases, and hence, the ROE decreases.

### 5.1 Effect on ROE

The success of the company as determined by a reliable variable Table-3 presents the results of ROE's five different regression models. The regression model with respect to the regression1, regression 2, regression 3, regression 4, regression 5 has an Adjusted R square value of 13.8 %, 12.3 %, 10 %, 11.7 % and 12.2 % respectively and F statistics of 11.02 %, 9.75 %, 7.972 %, 9.2.

67 and 8.466 % respectively. Based on the regression table, a significant positive value of F indicated the validity of the model. However, Low adjusted R squared value of Dequity, STD, LTD, TDTA means that the independent variable does not explain ROE, ROA and EPS completely. Therefore, the variable that can explain the remaining percentage are not included in our study.

**Table 3:** Effect on ROE.

Dependent Variable: ROE					
Independent Variables	Regression 1	Regression 2	Regression 3	Regression 4	Regression 5
	Beta	Beta	Beta	Beta	Beta
Dequity	0.019***				
STD		0.117***			0.116***
LTD			−0.050		−0.047
TDTA				0.100***	
Liquidity	0.000	0.001	−0.001	0.000	0.000
Gr_rate	0.041**	0.043**	0.044**	0.040**	0.044**
Inflation Rate	−2.096*	−2.003	−2.071	−1.934	−2.018
Ln (Tax)	0.040***	0.037***	0.039***	0.040***	0.037***
Firm Size	−0.04***	−0.033***	−0.035***	−0.036***	−0.032***
R Square	0.152	0.137	0.114	0.131	0.138
Adjusted R Square	0.138	0.123	0.100	0.117	0.122
F-score	11.029	9.759	7.972	9.267	8.466
P-Value	0.000	0.000	0.000	0.000	0.000

***, **, *, Dequity. STD, LTD, TDTA and ROE simultaneously indicate the level of significance at 1 %, level significance at 5 %, level significance at 10 %, debt-equity ratio, short term leverage, long term leverage, total leverage and return on equity.

The beta coefficient of Dequity is 0.019 and thus has a positive influence on the ROE indicates that 1 unit of Dequity will increase ROE by 0.019 when other variables remain constant. We have found t value of Dequity is significant at 1 % level. The result is showing a positive relationship between Dequity and the ROE. This result is consistent with Saputra, Noer, and Lukytawati [32], Vatavu [22], who found a positive relationship between them.

Furthermore, when the equity reduces in a firm, the Dequity increases, and so does the return on equity. There is an inverse relationship between equity and ROE. Similarly, according to the Miller and Modigliani theory, debt is tax-deductible, and the savings from tax increase return to the shareholders. So, there is a positive relationship between Dequity and ROE. However, Kamara et al. [70] argued that when debt increases, the shareholders demand excess return because, in the case of bankruptcy, the shareholder will face the ultimate loss. Hence, the increased level of debt ultimately requires more return to the shareholder. Thus, this ultimately fails to provide an expected rate of return to the equity holder in many extents, even if the ROE increases.

The regression between STD and ROE, showing a coefficient of 0.117. So, 1unit of STD will increase ROE by 0.117. The beta coefficient is showing a positive relation between STD and significant at 1 % level. Similar results found in the study of Abor [71] on the Ghana Stock exchange, where he found a significant positive relationship between the short-term debt ratio and ROE and explained that STD low-interest expenses help firms to increase their profit. Therefore, increasing STD also increases ROE. But this result is inconsistent with Vatavu [22], Shubita, and Jaafar [72]. They explained that the STD interest rate is relatively higher than LTD, so if firms fail to utilize the fund, they will face financial distress.

The regression between LTD and ROE shows an insignificant relationship with negative aspects. The coefficient of LTD is – 0.050. So, 1 unit of LTD will decrease ROE by 0.050. Due to the long-term debt, the company needs to pay more interest expenses, which reduce the net profit of the firm as well as ROE. So, we observe LTD's negative influences on ROE here. A similar result was found in the empirical research [33] conducted by Anowar [11], considering 40 manufacturing firms of Bangladesh in 2016. He also observed a negative relationship between return on assets and LTD and stated that the more long-term debt in the capital structure, the more fixed interest cost increases. Hence, the ROE decreases. This is also consistent with Fama and French [73], Graham [74], and Booth et al. [46] Azhagaiah and Gavoury [75].

A significant positive relationship can be observed between ROE and TDTA at 1%with a co-efficient of TDTA is 0.100. When an organization uses a higher level of the debt in the capital structure, it increases the total leverage level, in case the asset is fixed. Therefore, increasing total leverage increases the ROE, which shows a positive influence of TDTA on ROE. But Vatavu [22] came up with different result he explained that more the firm deploy debt it becomes less profitable.

The effect of control variable liquidity has a low level of influence on the return on equity. The research conducted by Rakesh and Souza [76] also found a low level of effect of liquidity on ROE. This is because the ROE is measured considering net income, not liquid assets. There is a massive difference in an organization's cash-flow and net income because there may be a considerable level of deferred income that increases ROE but, a low level of cash might be in hand, and thus, liquidity has less effect.

Among the control variables, tax and firm size have a significant effect on ROE at a 1 % level of significance. However, the tax has a positive effect on ROE, where the firm size effects ROE negatively. Anowar [11] described the phenomenon in Bangladeshi firms as the increase in firm size through leverage increases the fixed interest expense as well as increases the cost of capital. So, the excess leverage decreases the ROE when the firm enhances its size using a high level of debt. Inflation has a negative relation with ROE but not significant, except in the case of Dequity.

### 5.2 Effect on ROA

The effect of Dequity, STD, LTD, and TDTA on ROA is shown in table-4. The adjusted R square of five regression is 13.9 %, 12.4 %, 15.7 %, 15.4 % and 15.5 % respectively F statistics of 11.13 %, 9.878 %, 12.709 %, 12.432 % and 10.68 % respectively. As F statistics are significant, this model is valid. However, the independent variable does not completely describe ROA because the adjusted R square value is considered low.

**Table 4:** Effect on ROA.

Dependent Variable: ROA					
Independent Variables	Regression 1	Regression 2	Regression 3	Regression 4	Regression 5
	Beta	Beta	Beta	Beta	Beta
Dequity	−0.006***				
STD		−0.001			−0.003
LTD			−0.099***		−0.099***
TDTA				−0.062***	
Liquidity	0.001	0.001	0.001	0.000	0.001
Gr_rate	0.020**	0.019**	0.022**	0.021**	0.022**
Inflation Rate	−0.993*	−1.006*	−1.037*	−1.080*	−1.038*
Ln (Tax)	0.023***	0.023***	0.022***	0.023***	0.022***
Firm Size	−0.02***	−0.025***	−0.022***	−0.024***	−0.022***
R Square	0.153	0.138	0.171	0.168	0.171
Adjusted R Square	0.139	0.124	0.157	0.154	0.155
F-score	11.131	9.878	12.709	12.432	10.868
P-Value	0.000	0.000	0.000	0.000	0.000

***, **, *, Dequity. STD, LTD, TDTA and ROA simultaneously indicate the level of significance at 1 %, level significance at 5 %, level significance at 10 %, debt-equity ratio, short term leverage, long term leverage, total leverage and return on asset.

We have found the beta coefficient of the Dequity is −0.006, with significantly negative at a 1 % level. The negative relationship with the Dequity and ROA is showing that an increase in Dequity will reduce ROA. This means when Dequity increases, the company's total debt also increases, which requires the company to pay more interest on the debt, and therefore, the amount of net profit reduces, and so is the ROA. Therefore, increasing Dequity reduces ROA, which shows a negative influence. A similar result was found in the research of Anwar [77] and stated that when the debt level increases, it requires increase interest payment, and hence the overall ROA decreases. But this result is inconsistent with Rub [37], Sayeed [15] because a higher debt will provide a higher return as well as higher firm value.

We have found that the beta coefficient of STD is −0.001. So, 1 unit of STD will decrease ROA by 0.001. The negative but insignificant relationship between STD and ROA has been identified. Sayeed [15] Hossain and Ali [78] and Siddiqui [79] found similar results. When an organization uses STD, it keeps its cash to themselves, and this ultimately reduces the outflow. This hits the bottom line and thus decreased ROA. So, our result is quite different from the previous studies. Moreover, this could be due to short-term debt-related interest rate costs that could decrease return on assets. But [16,80], found a positive relationship between STD and ROA in DSE listed companies because operating in short-term debt for the operation reduces the long-term financing cost of operation, which ultimately increases ROA.

The co-efficient of LTD is −0.099, which shows that LTD has a negative relationship with ROA at a 1 % level. So, when LTD is increased by 1unit, the ROA will be decreased by 0.099. If we increase the long-term debt than interest, the expense will also increase. Thus, it will reduce the net profit and ROA. So, the negative influence is easily perceived. The research of Rakesh and Souza [76], Ikapel and Kajirwa [41] also found a negative relationship between LTD and ROA because the higher level of long-term debt demands high-interest payment obligation and this ultimately reduces the net income and hence, a negative effect on ROA. The pecking order theory also suggests using internal finance than the external due to the increased cost of using. So, the reduction in the net income and thus the reduction in the ROA could be described by this.

The beta coefficient of TDTA is −0.062. We have found the value of TDTA is negatively significant at a 1 % level. This is stating that an increase of 1 unit in the TDTA will decrease the ROA by 0.062. Similarly, Vatavu [22], Saputra, Azam, Lukytawati [32] found a negative relationship between TDTA and ROA. When an organization uses the external funds to invest in new ventures, the interest outlay increases, and this ultimately reduces the ROA as net income is calculated after deducting the interest paid. Besides, Ming-Chang Chang and Zuwei-Ching Tzeng [81] found a significant positive relationship. They explained that according to agency cost theory, there is a positive relationship among long term debt ratio and firm performance as debt cost will reduce the agency cost.

Again, the control variable's liquidity hardly influences the return of the firm. The research of Anowar [11] also found an insignificant effect of liquidity on profitability because financials operate accrual basis. Thus, liquid asset only creates a little effect on ROA.

Considering the control variables of the regression model, we have found that the firm size and tax have a significant effect and serious impact on ROA. This is because using debt provides a tax shield that increases the net income; thus, the ROA, according to Akhtar et al. [82]. The firm is showing a significant negative relationship with ROA at a 1 % significance level. A similar result was found by Anowar [11]. It stated that when an organization expands its assets, it takes a high level of leverage, which increases interest expense as well as the cost of capital. Thus, a negative relationship between ROA and firm size pertains. Besides, we have also found the gr_rate as a significant predictor of ROA at a 5 % level of significance. This is because the increase in sales increase the net profit and thus increase ROA and vice-versa. The research conducted on U.S. S & P 500 firms by Martis [49] also found the firm size and gr_rate as a significant factor influencing ROA but, he did not find a tax as a factor that influences ROA.

### 5.3 Effect on EPS

In < Table 5> firm performance measured by dependable variable EPS have five regression model. The regression model with respect to the regression1, regression 2, regression 3, regression 4, regression 5 has an Adjusted R square of 23.9 %, 26.1 %, 24.4 %, 24.4 %, 26.4 % respectively and F statistics of 20.67 %, 23.098 %, 21.265 %, 21.197 %, and 20.24 % respectively. The adjusted R square indicates 76.1 %, 73.9 %, 75.6 %, 75.6 %, and 73.6 % of the variance in this table can be predicted from independent variables. In contrast, the remaining influenced by others which are not considered for this study. Based on the regression table, a significant positive value of F indicated the validity of the model.

**Table 5:** Effect on EPS.

Dependent Variable: EPS					
Independent Variables	Regression 1	Regression 2	Regression 3	Regression 4	Regression 5
	Beta	Beta	Beta	Beta	Beta
Dequity	−0.009				
STD		1.276***			1.265***
LTD			−0.964*		−0.929
TDTA				0.599	
Liquidity	−0.021	0.000	−0.022	−0.010	−0.002
Gr_rate	0.217	0.215	0.242	0.199	0.240
Inflation Rate	−23.600*	−23.051*	−23.936*	−22.901*	−23.360*
Ln (Tax)	0.552***	0.527***	0.544***	0.554***	0.519***
Firm Size	−0.145	−0.117	−0.125	−0.152	−0.095
R Square	0.251	0.272	0.256	0.256	0.277
Adjusted R Square	0.239	0.261	0.244	0.244	0.264
F-score	20.676	23.098	21.265	21.197	20.246
P-Value	0.000	0.000	0.000	0.000	0.000

***, **, *, Dequity. STD, LTD, TDTA and EPS simultaneously indicate the level of significance at 1 %. At the same time, this represented that level significance at 5 %, level significance at 10 %, debt-equity ratio, short term leverage, long term leverage, total leverage and earnings per share.

Table 5 shows the effect of the dependent variable on the EPS. The beta coefficient of Dequity is −0.009 on EPS. 1 unit increase in Dequity will reduce the EPS by 0.009. Dequity is not significant at a 1 % level. When Bangladeshi firms increase debt, the firm's net profit reduces due to the increased level of interest expense, which ultimately reduces the EPS as well. So negative influence has been found. A similar result was found in the study of Le and Phan [83], where they opined that the interest paid to the equity holder reduced the net income, and thus, the EPS reduced as the total debt level increased. Anowar [11] also observed that in Bangladeshi firms, a relation between decreases in profit with the increase in debt due to a reduction in the net profit with an increased level of interest payment.

The coefficient of STD is 1.276. So, an increase in short term debt by 1 unit, EPS will increase by 1.276 while other variables remain constant. We have found that the result is positively significant at 1 %. Rakesh and Souza [76] found a similar result. They stated that when an organization has short-term debts, it has a greater income because it enhances the net income without increasing the fixed interest payment. But Mahfuzah Salim, Dr. Raj Yadav [84] found a negative relation between STD and EPS at a 1 % significant level. Because when a firm uses too much debt, the organizational structure becomes volatile.

The coefficient of LTD is −0.964. It states that an increase in LTD by 1 unit will decrease the EPS by −0.964 point, and it is negatively significant at a 10 % level. Increasing long term debt increases interest expense and thus reduces the net profit and EPS. So, the negative influence is easily perceived. According to Akhtar et al. [82], the more the organization uses LTD, the fixed interest cost increases, and hence, it decreases the net profit and thus the EPS. So, the right balance of debt and equity could ensure the profitability of the organization.

We have found the beta coefficient of TDTA 0.599, which means an increase in TDTA by 1 point will influence the EPS for increasing by 0.599 points. This is because leverage is mainly taken by organizations to fund new financial endeavors, and hence the growth of financial increases the net profit and thus the 10.13039/100012086EPS. A similar result was found in the study conducted by Anowar [11], where he stated that leverage provides the opportunity to grow financial and levers profitability. Hence, the right mix of debt and equity could improve EPS. Besides, he referred Miller and Modigliani hypothesis II, where he stated that debt provides tax shield, which increases the net income and thus the EPS. On the other hand, Salim, M. et al. [85] found a negative relation between TDTA and EPS. He explained that the more the organization uses debt, the interest cost increases and finally reduce profit [86].

Considering the control variables gr_rate, inflation rate, tax, and firm, the tax is a significant effect on EPS on a 1 % level. This is because debt is tax-deductible, and higher tax provides a tax shield, which ultimately increases the EPS of the shareholders. According to Akhter et al. [23], Miller and Modigliani proposition two suggests the tax shield advantage from using debt increases the firm value. Hence, a positive relationship could be found between EPS and tax payment. The firm size negatively affects EPS. However, it is not significant. But the firm size has a negative and non-significant effect.

Finally, based on the above analysis STD, TDTA shows a significant positive relation with ROE. ROE is calculated by dividing net income divided by equity. So, if equity decreases ROE will increase. Moreover, from descriptive analysis, we know that Bangladeshi firms prefer debt than equity so a positive impact of STD and TDTA is consistent. Similarly, Saputra, Azam, and Lukytawati [32], Arabahmadi and Arabahmadi [87], Abor [71] found a positive relation between Debt ratio and ROE. They described that debt has an extra tax shield opportunity. When a firm use debt due to the tax-saving nature of debt net profit increased. As a result, ROE increase. On the contrary, Bokhtiar, A. F. et al. [88] found a negative relation between Debt ratio and ROE. Similarly, Ebaid [89], Vatavu [22], Ali and Iman [90], and Zeitun and Tian [91] found a negative relation between ROE and debt ratio. They explained that due to a higher cost of debt and tax incident they found a negative relationship between debt ratio and ROE [92].

However, we found a negative relationship between all the debt ratios and ROA. This is consistent with the previous study of Md. Bokhtiar, A. F. M. Mainul, Md. Afzalur & Md. Nurul [93] and Rouf [16], Zahid Anowar [11] in Bangladesh. They explained that when an organization uses external funds, interest outlay increases. This ultimately reduces the ROA as net income is calculated after deducting the interest paid. Moreover, with a higher level of debt, interest expenses also increased as a result ROA decreased. Similarly, Saputra, Azam, and Lukytawati [32] in Indonesia, Vatavu [94] in Romania and Abeywardhana [95] in the U.K. found a negative relationship between debt ratio and ROA. They stated debt ratios interest expenses decreased profit. Consequently, firms ROA decreased. On the other hand, Abor [71] in Ghana, Ming-Chang Chang and Zuwei-Ching Tzeng [81] in China, Ozketin [96] in several countries found a positive relationship between debt ratio and ROA. They described that the firms having a level of leverage in the capital structure, they must need to perform well to meet the demand of the shareholders and lenders.

The most important finding in our research is long term debt has a negative relationship with all the performance measures ROA, ROE, and EPS. Similarly, Abor [71] in Ghana, Rouf [97] in Bangladesh, Ikapel and Kajirwa [98] in Kenya, Shubita and Jaafer [99] in Amman stock Exchange found a negative relationship between LTD and Firm performance. This result is also consistent with Fama and French [73], Graham [74], Booth et al. [46] and Azhagaiah and Gavoury [75]. They stated that long term debt interest expenses reduced profit margin which ultimately reduce firm performance. This result is also supported by the pecking order theory. Because according to, pecking order theory firms first choose debt than equity. But a higher proportion of debt leads a firm to low profitability due to interest expenses as a result profitability decrease.

Control variable Firm size has a negative effect on all firm the firm performance proxies. Anowar [11] found a similar result and explained that when a firm expands, it takes a high level of debt as a result of interest expenses increase and consequently cost of capital increase. On the other hand, Tax has a positive effect on ROA, ROE, and EPS. Because debt provides tax shield as a result net income increased. Akhtar et al. [82] found a similar result in Bangladesh. However, the control variable inflation rate has a negative effect on all firm performance proxies. Similarly, Roberts et al. [53], Martis [49] found a negative relationship between firms’ performance and inflation. They described that inflation reduces the value of the return and consequently decreases buyer purchasing power and firm performance. Moreover, the Growth rate has a positive impact on all firm performance proxies. Martis [49] in the U.S.A, Anowar [14] and Rouf [16] in Bangladesh, Abor [100] in Ghana, Dr. Shubita & Dr. Jaafer [101] and Ajayi and Zahiruddin [58] found a positive relationship between growth rate and firm performance proxies. They stated that growth rate increases the profit of the firms and thus profitability increases consequently firm performance increase.

## 6 Conclusions

The research scrutinized the impact of various firm-specific factors on the capital structure and subsequent profitability of Bangladeshi firms. Utilizing panel data encompassing 78 companies listed on the DSE from 2017 to 2021, determinants of capital structure included debt to equity ratio, short-term and long-term leverage ratios, liquidity ratio, and total debt ratio. In parallel, profitability was measured through Return on Assets (ROA), Return on Equity (ROE), and Earnings Per Share (EPS), with additional control variables like inflation rate, growth rate, tax rate, and firm size. Descriptive analysis revealed a preference for short-term debt over long-term debt among Bangladeshi firms, aligning with the objective of wealth maximization. Correlation analysis indicated moderate correlations of total debt ratio with other independent variables, except for liquidity. Notably, ROA and ROE showcased a strong positive correlation, in line with findings from Islam et al. [102] in Pakistani manufacturing firms. Regression models tested three dependent variables against four independent and five control variables. The analysis demonstrated significant positive effects of Dequity and short-term debt (STD) on ROE at a 1 % significance level. Conversely, long-term debt (LTD) exhibited a negative and insignificant impact on ROE, aligning with findings by Saputra, Noer, and Lukytawati [32], and Vatavu [22] who highlighted adverse effects of high long-term debt on profitability.

Regarding ROA, Dequity, STD, LTD, and total debt ratio (TDTA) showed negative and significant effects at a 1 % level, signaling a potential decrease in net profit and return on assets with increased debt levels. However, findings contrasted with studies by Sayeed [15] and Rub [37], underscoring divergent interpretations of the impact of debt on profitability. In analyzing EPS, STD exhibited a significant positive impact at 1 %, whereas LTD demonstrated a negative significance at 10 %, contrary to the hypothesis. Notably, Dequity showcased a positive sign but lacked significance, resonating with Akhter et al.'s (2016) findings that EPS predominantly depends on net income rather than liquidity. The analysis highlighted several significant findings, notably the positive relationship between certain variables like STD and ROE, while also underscoring negative impacts on ROA and EPS with increasing debt levels. Moreover, variables like sales growth, inflation rate, tax rate (Ln Tax), and firm size showcased significant effects on ROA and ROE, corroborating with Anowar's [103] research.

Overall, the study establishes a significant link between capital structure and financial performance in Bangladeshi firms, aligning with findings by Gleason et al. [104], Arbor [105], Hussain et al. [106], Gill et al. [107], and Micah et al. [108]. The varying interpretations emphasize the multifaceted nature of debt's influence on firm performance, ranging from increased costs of capital due to higher debts to positive signals perceived by shareholders. Moreover, the Miller & Modigliani theory underscores the role of tax shields in enhancing profitability [20].

### 6.1 Policy recommendations

According to the results of this research, one may draw the conclusion that having a capital structure that is comprised of a significant amount of long-term debt will result in below-average financial performance for a firm. According to the findings of this research, the influence of the tangibility of assets and cash flows on the primary financial performance indicators of cement companies yields inconsistent results.

As a whole, the accounting performance of Bangladeshi Companies has been persistently poor throughout the course of the years, as shown by the measures that have been presented. These findings imply that managers of companies need to exercise extreme caution when determining how much long-term debt to incorporate in their capital structure, since doing so may have a major and detrimental impact on the bottom line of the business. The subject of how the company's financial structure affects its performance is a vital one that needs an answer as soon as possible and must be resolved without delay. For the sake of human health over the long run, we need further study into this subject area.

### 6.2 Future recommendations

The research has considered only 78 Bangladeshi firms of DSE, but there is 586 firm listed in DSE. Similar studies on Bangladesh firms and the capital structure were conducted by [103,109,23] took less than 100 firms in their study. So, the recommendation for future research is to increase the firm size to understand the effect of capital structure on firm profitability better. As this study found some independent variables as a not significant influencer of profitability, hence, increasing the sample size could provide a better understanding of these variables. This research does not consider all the factors determinants of firm performance.

The low adjusted R square value indicates that our independent variable does not explain the dependent variable completely. So, adding other variables could lead to other outcomes. Further studies can be conducted by adding financial risk and tangibility as the independent variables. Besides, the managers of organizations need to determine the optimum capital structure by reducing the overall cost of capital and increase the profitability or the return to the shareholders. Moreover, this research does not consider proxies of profitability measure that consider liquidity. So, the recommendation for further research is to consider the measures that count liquidity. Also, this research only considered a non-financial organization. Further research can be conducted by adding both the financial and non-financial sector with comparative analysis.

### 6.3 Limitations

It is crucial to note that our research contains a number of limitations. It started by gathering all of its data through annual reports, which is referred to as secondary data. It is recommended that future research mix primary data (such as data gathered via surveys or interviews) with secondary data (such as data from financial archives). The conclusions of this study, which was conducted in a developing country and only covered a limited number of listed companies, cannot be applied to other industries or financial conditions. More research that compares this problem to more developed nations and includes samples from a larger variety of sectors is needed to fully understand it.

### 6.4 Implications

The implications of these results suggest that managers should exercise considerable care when making decisions on the optimal level of long-term debt to include in their company's capital structure. This is due to the potential for significant and adverse effects on the financial performance of the organizations.

## Data availability statement

Data will be made available on request.

## Funding

This research received no external funding.

## Additional information

No additional information is available for this paper.

## CRediT authorship contribution statement

**Fahad Ahmed:** Writing – original draft, Resources, Investigation, Formal analysis, Conceptualization. **Mujib Ur Rahman:** Writing – review & editing, Visualization, Resources, Investigation, Data curation. **Hafiz Mudassir Rehman:** Visualization, Validation, Resources, Methodology, Investigation, Formal analysis, Data curation. **Muhammad Imran:** Visualization, Validation, Software, Resources, Investigation, Data curation. **Anna Dunay:** Writing – review & editing, Validation, Resources, Project administration, Methodology, Investigation, Funding acquisition, Conceptualization. **Md Billal Hossain:** Writing – review & editing, Validation, Supervision, Methodology, Investigation, Funding acquisition, Formal analysis, Data curation, Conceptualization.

## Declaration of competing interest

The authors declare that they have no known competing financial interests or personal relationships that could have appeared to influence the work reported in this paper.
